# Measuring Difficulties in Choosing an Upper Secondary School: Validating of the Parental Expectations, Confusion, Anxiety and Suitability Scale (PeCAS Scale)

**DOI:** 10.5964/ejop.v16i4.1812

**Published:** 2020-11-27

**Authors:** Diego Boerchi

**Affiliations:** a Faculty of Education, Department of Psychology, Catholic University of the Sacred Heart, Milan, Italy; University of Wroclaw, Wroclaw, Poland

**Keywords:** educational guidance, difficulties in career choices, career exploration, parental expectations, secondary school students

## Abstract

This study was aimed to develop a new questionnaire, on school choice difficulties, with a limited number of items and scales to make it suitable both for pre-screening on large numbers of students and studies which use batteries of many tests. The PeCAS Scale assesses four dimensions, Parental expectations, Confusion, Anxiety, and Suitability, which could be considered the most essential according to previous literature. In total, 1495 students participated in the study. Both exploratory and confirmatory factor analysis supported a four-dimensional factor structure. Reliability and concurrent validity, concerning the process of choosing a school and a scale on career exploration, were also adequate.

The choice of an upper secondary school is a difficult one, and all students are not mature enough or do not have access to adequate support to take on such a task (Howard & Walsh, 2010). In Italy, this choice is more challenging because it has to be made when they are 13-14 years old and students have to choose between a wide range of schools (6 lyceums, with general topics and a focus on one of them like sciences, arts or languages; 11 technical institutes, very focused on technical topics like economics, tourism or informatics; 6 professional institutes and 21 professional qualifications, very focused on specific operative jobs like mechanic, cook or hairdresser). Unfortunately, both schools and families tend to invest limited resources in these kinds of activities: Sometimes they underestimate the importance and impact of this choice, with negative consequences such as school failure or strong dissatisfaction and poor performance of the students. On the one hand, it can be useful to enable targeted counselling aimed both at students, to help them to understand their psychological characteristics better and to collect information about educational prospects, and to their teachers and parents, so that they can support them (Boerchi, D’Urso, & Pace, 2019). On the other hand, not all students need to be supported and, to optimise the use of limited resources, it could be useful to identify those with greater difficulties to intervene in a targeted and prompt manner.

The Anglo-Saxon language differentiates between "indecision", indicating an early and normal stage of any decision-making process and therefore a common experience in the life of every individual in relation to career choices (Germeijs & De Boeck, 2002; Germeijs & Verschueren, 2007), and "indecisiveness", which "refers to a chronic inability to make decisions in different contexts and situations" (Di Fabio, Palazzeschi, Asulin-Peretz, & Gati, 2013, p. 43). In this second case, it is essential to understand how specific personality characteristics can affect the process of choice, making it difficult. Literature, for example, showed that negative relationships exist with extroversion and emotional intelligence and positive relationships exist with introversion (Di Fabio & Palazzeschi, 2009; Di Fabio et al., 2013). But even if the problem cannot be traced back to a personal and structural limit on decision making, you can still identify a condition of difficulty related to personal or contingent factors that make career choices more difficult. According to Gati and Levin (2014), there are many factors that, combined, make a career choice more complex and difficult: the number of alternatives to choose from is becoming wider and wider; more and more factors should be taken into account (e.g. the employment context, length of training, choosing to use specific skills such as one's numeric aptitude); there is always greater uncertainty about both themselves and the world of work; most career decisions require more compromises than in the past; customers tend to limit their choices because of social barriers, both real and imaginary; finally, customers are increasingly aware of the importance of their decision and they worry about making the "wrong" choice. It is therefore important to understand if a lack of decision is due to an early stage of decision making, otherwise called "developmental indecision" (Guay, Ratelle, Senécal, Larose, & Deschênes, 2006; Osipow, 1999; Tinsley, 1992;), or whether it is a structural element of the personality of the individual (Saka & Gati, 2007; Saka, Gati, & Kelly, 2008). Another possibility could be that it is just becoming a critical issue because of specific difficulties that need to be identified and thus addressed (Osipow, 1999).

The international literature provides us with different questionnaires, the purpose of which are to investigate what kind of difficulties you are encountering in the process of choosing a training course or a job. Concerning indecisiveness, the most suitable questionnaire is the Emotional and Personality- Related Career Decision-Making Difficulties (EPCD; Saka & Gati, 2007; Saka et al., 2008; 53 items). It explores 11 personality traits that may cause chronic career indecision like: pessimistic views (about the process, about the world of work, about one’s control); anxiety (about the process, about uncertainty, about the choice, about the outcomes); self-concept and identity (general anxiety, self-esteem, uncrystallized identity, conflictual attachment and separation). The authors found few gender differences and with small effect size: just for the scale of anxiety about the process (Cohen’s *d* = 0.17), where females had greater difficulties, and for the scales of pessimistic view about the process (*d* = 0.29), where males had greater difficulties (Saka et al., 2008).

Concerning indecision, Kelly and Lee (2002) argue that it is possible to identify three approaches, represented by the same number of questionnaires. The first approach is represented by the Career Decision Scale (CDS; Osipow, 1987), developed from the clinical experience of the author and his colleagues, which is particularly effective in identifying changes related to targeted interventions. The main limitation of this tool is the uncertainty concerning its dimensionality. According to the author, it consists of a scale on certainty, including two items, and of a scale on indecision, comprising of a 16 items scale. But, concerning the scale on indecision, Laplante, Coallier, Sabourin, and Martin (1994) and Martin, Sabourin, Laplante, and Coallier (1991) argued that it is one-dimensional considering just 6 items; Kelly and Lee (2002) proposed a three-dimensional model comprising of 11 items (identity diffusion, positive choice conflict, tentative decision); Schulenberg, Shimizu, Vondracek, and Hostetler (1988) and Shimizu, Vondracek, Schulenberg, and Hostetler (1988) identified four factors which include 16 items (negative confusion, positive confusion, desire for support, internal/external barriers). CDS was adapted into an Italian context (Nota, Ferrari, Solberg, & Soresi, 2007) on high school students, but its structure was not understandable on this occasion. Authors, as a first step, found a four-factor structure, two of them analogous to those proposed by Osipow (1987) and, in the end, they decided to average the 16 items as a global index of career indecision: The Cronbach alpha coefficient was .81, and no differences were found between males and females in University-Preparation School. The second approach is represented by the Career Factors Inventory (CFI; Chartrand, Robbins, Morrill, & Boggs, 1990) composed of 21 items and based on the idea that there are two broad categories of problems related to decision making: Lack of information and emotional impediments to the completion of the decision-making process. Exploratory and confirmatory factor analyses confirmed a stable structure of four factors: The need for career information; the need for self-knowledge; anxiety related to a career choice; general indecision (structural). The only significant gender difference was on the Generalized Indecisiveness Scale, *t*(313) = 2.75, *p* < .01, with women describing themselves as more indecisive. CFI was adapted into an Italian context (Lo Presti, Pace, Lo Cascio, & Capuano, 2017) and tested on students attending high school and university: The Cronbach alpha coefficients ranged from .87 to .64. The third approach is represented by the Career Decision-Making Difficulties Questionnaire (CDDQ; Gati, Krausz, & Osipow, 1996) a 44 item questionnaire developed to test a theoretical taxonomy on the difficulties in career decision-making consisting of 3 categories and 10 subcategories: Lack of readiness (lack of motivation, general indecisiveness, and dysfunctional beliefs); lack of information (about the career decision-making process itself, the self, occupations or majors, and ways of obtaining additional information and help) and inconsistent information (unreliable information, internal conflicts, external conflicts). The authors reported Cronbach alpha coefficients for Israeli and American samples ranging from .63 to .95. Despite the wider range of difficulties considered, this questionnaire is limited to cognitive aspects, completely omitting the role of anxiety, as well as no-optimal levels of internal consistency in some scales. CDDQ was also adapted into an Italian context (Di Fabio & Palazzeschi, 2013) and tested on high school and university students: The Cronbach alpha coefficients ranged from .87 to .70 and results were similar for women and men.

None of the questionnaires considers the role of parents in choosing a school. According to the social cognitive career theory of Lent, Brown, & Hackett (1994), parents represent one of the most important Contextual Influences Proximal to Choice Behavior. On the one hand, they can provide support; on the other hand, their expectations can influence the choice negatively (Boerchi & Tagliabue, 2018). Schmitt-Wilson (2013) found that “perceiving parental expectations, along with the control variable of gender, significantly explained educational expectations” of the students (p. 234). Shen, Liao, Abraham, and Weng (2014) found, on a sample of Asian Americans, that living up to parental expectations influences self-efficacy directly in stereotypical occupations, interests in stereotypical occupations and outcome expectation in stereotypical occupation, and it mediates between these and parental pressure. The limit of these and similar studies is that parental expectations are limited to the intended educational level of child (Andres et al., 2007) or academic achievements (Wang & Heppner, 2002), not to the parental expectations of choosing a specific school, which can be in contrast with child desires producing difficulties in the choice making.

## The Present Study

Some of the questionnaires on difficulties in choosing a school described here feature many items and factors, others have some psychometric limits, and all of them were developed on samples of high school or university students. For an operational use directly managed by teachers and for studies based on the use of a battery of questionnaires, a new scale would be useful featuring by: the representativeness of the most common psychological dimensions highlighted in the previous literature; a small number of items and factors; valid to be used with students from 13 years and older; simple to administer and interpret for teachers and non-psychologist counsellors.

The aim of this study, then, was to draft and seek evidence of the reliability and structural validity of a new questionnaire about school choice difficulties by:

1. Drafting the questionnaire structure starting from the previous literature;
2. Testing the latent factor structure through both exploratory and confirmatory factor analysis;
3. Providing evidence regarding the internal consistency of all the subscales through Cronbach's α;
4. Providing evidence regarding the concurrent validity of the subscales by testing the hypothesis of positive relation with a scale on an exploration of vocational issues;
5. Providing evidence regarding the concurrent validity of the subscales by testing the hypothesis of positive relation with the number of options the students are considering.

## Method

### Participants

The research involved 36 middle schools, composed of one to five groups of students. Schools were in a specific region in the north of Italy with middle and small cities, characterised by having a very wide, but not complete, range of upper secondary schools and some problems of transportation which partially reduce students’ choice. In total, 1495 students, attending the last year, answered the questionnaire. They corresponded to near the whole population of the region: Just a few of them didn’t participate at the study or because not present at school, or because of the presence of severe physic or cognitive disabilities. 50.6% were male, and 49.4% were females. Participants’ mean age was 12.93 (*SD* = 0.624), ranging from 11 to 15 (73% were 13 years old).

### Procedure

The administration took place in November, two months after the beginning of the school and three months before the deadline of the enrolment to the upper school. Because of the aim to provide teachers with an instrument they can use directly, they were asked to administer the paper-pencil version of the questionnaire to their students in the classroom. Previously, teachers were provided with clear instructions on how to administer the questionnaire and how to manage students’ possible questions avoiding steering their opinions. The administration was made exclusively after parents’ authorisation and only after clarifying that no one student should feel obligated to participate.

### Measures

#### PeCAS Scale

The conception of the questionnaire was to draft a scale characterised by simplicity and inspired to the previous literature. Therefore, to design it, four psychologist experts in vocational guidance were involved in the study. They were asked to analyze separately the explanations of each scale of the three questionnaires previously described, CDS (Osipow, 1987); CFI (Chartrand, Robbins, Morrill, & Boggs, 1990); CDDQ (Gati, Krausz, & Osipow, 1996), and to group the scales for the similarity of the psychological variable assessed without any limitation about the number of clusters. Later, they met and agreed on the presence of two main clusters (information and indecisiveness) and two independent scales related to anxiety and barriers (Table 1).

**Table 1:** Analytic Classification of Psychological Dimensions Assessed in the Literature

Area	Career Decision Scale^a^	Career Factors Inventory	Career Decision-Making Difficulties Questionnaire
Information	Desire for support	Need for career information; Need for self-knowledge	Lack of information; Inconsistent information
Indecisiveness	Negative confusion; Positive confusion	General indecision (structural)	Lack of readiness
Anxiety	-----	Anxiety related to career choice	-----
Barriers	Internal/external barriers	-----	-----

^a^For the Career Decision Scale, the model of Shimizu, Vondracek, Schulenberg, and Hostetler (1988) was used.

This taxonomy was the base to develop the new questionnaire, which was drafted to be simple and representative of the four areas of difficulty a student can encounter, but not necessarily exhaustive about all the criticalities connected to school choice. It was composed of the following four subscales:

##### Pe – Parental expectations

Starting from all the barriers a student can encounter, it was decided to concentrate on just one of them that particularly affect the students’ choices. Sometimes students feel parents attempt to control too much of their actions and choices about their school. If this happens, they tend to be more confused and passive in the process of career preparation (Dietrich & Kracke, 2009). The first scale aims to assess if the student feels free or under pressure to respect parents’ expectations.

##### C – Confusion

This scale aims to assess the level of the indecisiveness of the student asking if he/she doesn’t feel able to choose because of the wide range of opportunity he/she likes.

##### A – Anxiety

This scale aims to assess the level of anxiety the student is feeling because of choice itself or the risk of making a mistake.

##### S – Suitability

This is the most complex scale of the questionnaire. It refers to the process of identifying the school, which better fits students’ expectations and abilities. It assesses the feeling of being able to gather information about the self, about the schools and to understand which one can be more suitable to his/her features.

Each subscale was composed of four items which were drafted selecting and adapting those more congruent with the scales’ content of the instruments previously described. Students is requested to indicate how much they agreed with each of them using a five-point Likert scale (1 = *Not at all*; 2 = *Slightly*; 3 = *Moderately*; 4 = *Very*; 5 = *Completely*).

#### Exploration of Vocational Issue Scale

It is plausible that those students who experience more difficulty in making a school choice will devote more time and energy in searching for information. A positive relation between exploration behaviours and the difficulties assessed by PeCAS can be considered a confirmation of its validity. It was decided to validate, in Italian, the Exploration of Vocational Issue Scale of Kracke (1997). It is a one-dimensional scale consisting of 6 items. In the original study (236 teenagers, mean age of 15.2 years and *SD* = .65), it showed a Cronbach’s α index of .70. First, all the items were translated and checked with the method of back-translation. Then, some items were partially modified to fit the specific choice of this sample of students better: for instance, “I talk to as many people as possible about occupations I am interested in” was modified into “I talk to as many people as possible about school courses I am interested in” (see Table 3). To simplify the task of the compilers, it was the same five-point Likert scale of PeCAS was used, instead of the four-point scale of the original version.

#### The Situation of the Choice

Another index that is supposed to be related to the difficulties in choosing a school is the situation of the students about choice. Those who have already chosen shouldn't feel in difficulty, while those who are still considering a wide range of opportunity is more likely are confused about the choice. Thus, a positive relation between the number of courses the student is considering, and the difficulties assessed by PeCAS is expected, and it can be considered a confirmation of the validity of the questionnaire. The situation of the choice was investigated by a single question, and students distributed quite homogeneously between the four alternatives: 1) “I have already decided which course to enroll” (*n* = 340, 22.7%); 2) “I am considering two alternatives” (*n* = 429, 28.7%); 3) “I am considering three alternatives” (*n* = 383, 25.6%); 4) “I am considering four or more alternatives” (*n* = 343, 22.9%).

## Results

Table 2 lists the PeCAS’ items and their psychometric characteristics: all of them showed to possess a clear normal distribution.

**Table 2:** PeCAS Items and Their Psychometric Characteristics

Code	No.	Item	*M*	*SD*	Skewness	Kurtosis
Pe_1	1	I would choose a course that my parents agree with.	2.60	1.04	0.32	-0.31
Pe_2	5	I think that parents are the most suitable people to say if a course is appropriate or not for their children.	2.72	1.05	0.24	-0.44
Pe_3	9	I would choose a course that does not disappoint my parents’ expectations.	2.53	1.00	0.30	-0.29
Pe_4	13	My parents' opinion is important to me about the courses that I am considering.	2.87	1.00	0.17	-0.27
C_1	2	Right now, I consider interest in the same manner of many courses.	2.64	1.06	0.31	-0.52
C_2	6	Since I have many interests, I find it hard to choose a course that I like more than all the others.	2.60	1.15	0.36	-0.68
C_3	10	I find many courses interesting that I can choose between.	2.99	1.00	0.03	-0.40
C_4	14	Although I know that finally I will have to choose just one, right now there are many courses that attract me.	2.72	1.18	0.31	-0.75
A_1	3	I don't feel calm when I think of the choice of studies I shall make.	2.67	1.16	0.30	-0.72
A_2	7	The idea that I could make the wrong choice of my studies gives me a little bit of anxiety.	3.09	1.21	-0.04	-0.90
A_3	11	I am concerned about the idea that soon I will have to choose which course to do next year.	2.71	1.16	0.26	-0.74
A_4	15	I am nervous when I look for information on courses that I could attend next year.	2.24	1.01	0.69	0.10
S_1	4	I do not know to gather reliable information on the courses I could attend next year.	2.45	1.00	0.46	-0.23
S_2	8	I do not know what are the aptitudes that are needed to face the courses I am considering.	2.88	1.00	0.07	-0.42
S_3	12	I do not know how to assess the reliability of the information provided to me about the different courses.	2.23	0.83	0.47	0.19
S_4	16	I can't understand in which courses I could be more successful.	2.83	1.01	0.13	-0.42

Also, all the items of the Italian validation of the Exploration of Vocational Issue Scale of Kracke (1997) adapted to school choices were normally distributed (Table 3). Confirmatory factor analysis (CFA) confirmed the one-dimensionality of the scale, χ^2^ = 48.610 (9), *p* < . 001; RMSEA = .054, CI [.040, .070]; CFI = .971, and Cronbach’s α was .72.

**Table 3:** School Version of the Exploration of Vocational Issue Scale’s Items and Their Psychometric Characteristics

No.	Item	*M*	*SD*	Skewness	Kurtosis
1	I talk to as many people as possible about school courses I am interested in	3.04	1.01	0.06	-0.41
2	I try to find out which subjects mostly interest me	3.92	0.86	-0.51	0.05
3	I try to get information about school courses I am interested in, in many possible ways (e.g., reading, talking, internships)	3.50	1.07	-0.31	-0.54
4	I try to find out which school courses best fit my strengths and weaknesses	3.63	0.94	-0.34	-0.21
5	When I seek information about a school course, I also try to find out about its negative aspects	3.23	1.03	-0.09	-0.53
6	I consider various school courses and try to get extensive information about all alternatives	3.29	1.02	-0.10	-0.49

### Construct Validity

To verify the structure of the questionnaire, the sample was randomly divided into two subsamples to test both the exploratory factorial analysis (EFA; with SPSS [Vesrion 25]) and the CFA (with AMOS [Version 25]) with the maximum likelihood method for both. The two subsamples did not differ significantly both for items‘ psychometrics and gender distribution. EFA was conducted on a sample of 748 students, and the number of factors to be extracted was defined comparing eigenvalue > 1 and scree plot criteria. Four factors were considered the best solution because eigenvalue of the 4th factor was lower but very near to 1 (.931), and screen plot suggested this solution. Factor loadings are reported in Table 4.

**Table 4:** EFA Factor Loadings

Item	1	2	3	4
A_3	**.862**	-.005	.031	-.030
A_1	**.798**	.013	.015	-.075
A_2	**.657**	.026	.034	.128
A_4	**.560**	.019	.061	.120
Pe_4	-.041	**.847**	.062	-.033
Pe_1	-.004	**.685**	-.044	-.033
Pe_2	.002	**.641**	-.041	.014
Pe_3	.068	**.515**	.048	.080
C_4	.006	-.042	**.852**	.067
C_3	-.100	.058	**.785**	-.036
C_1	.099	.030	**.714**	-.032
C_2	.110	-.080	**.691**	.052
S_2	-.033	-.023	-.027	**.806**
S_4	-.043	.098	.088	**.729**
S_3	.143	-.014	.038	**.364**
S_1	.221	.051	-.036	**.254**

*Note.* In bold are values bigger than .25.

The four factors explained 52.22% of the variance, with Factors 1 to 4 accounting, respectively, for 29.73%, 11.06%, 7.67%, and 3.21% of the variance. All factors included the same items of the original model with loadings greater than .50, except two items of the scale suitability.

**Figure 1:**
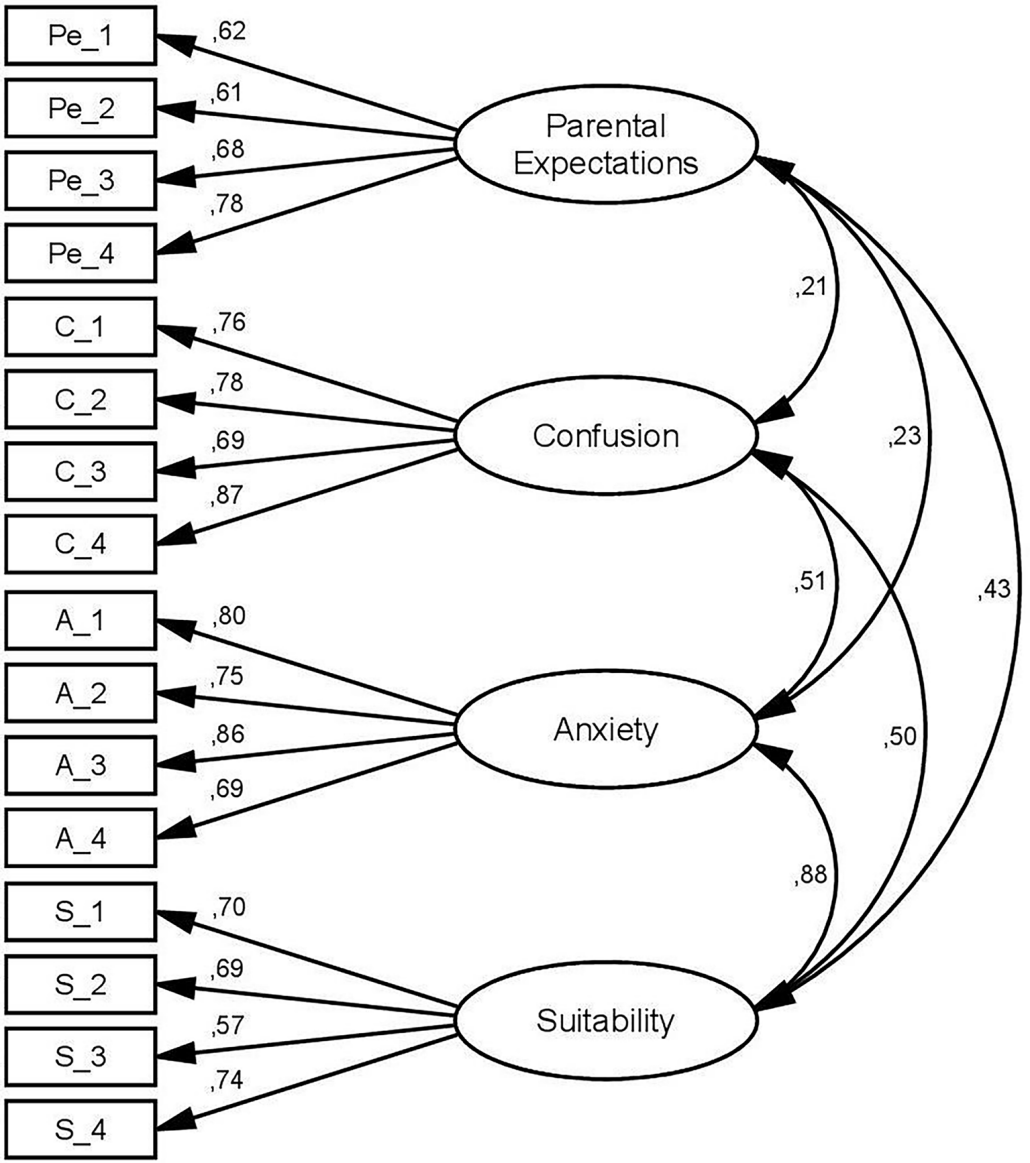
Structural Model of the PeCAS.

CFA was conducted on a sample of 747 students, testing a model consisting of 4 latent variables which affected four items each, and the correlations between the variables. Fit indexes confirmed the goodness of the model, χ^2^ = 326,787 (98), *p* < .001; RMSEA = .056 CI [.049, .063]; CFI = .956). Factors loadings were comprised between .57 and .86, and correlations between the factors were all positive and significant with *p* < .001 (see Figure 1 for details).

### Reliability

Internal consistency was estimated with Cronbach’s α on the whole sample and all indexes were good: parental expectations = .77; confusion = .86; anxiety = .85; suitability = .73.

### Concurrent Validity

All the scales of PeCAS correlated positively with career exploration, mostly confusion (*r* = .215, *p* < .001) followed by parental expectations (*r* = .151, *p* < .001), anxiety (*r* = .135, *p* < .001) and suitability (*r* = .105, *p* < .001).

**Figure 2:**
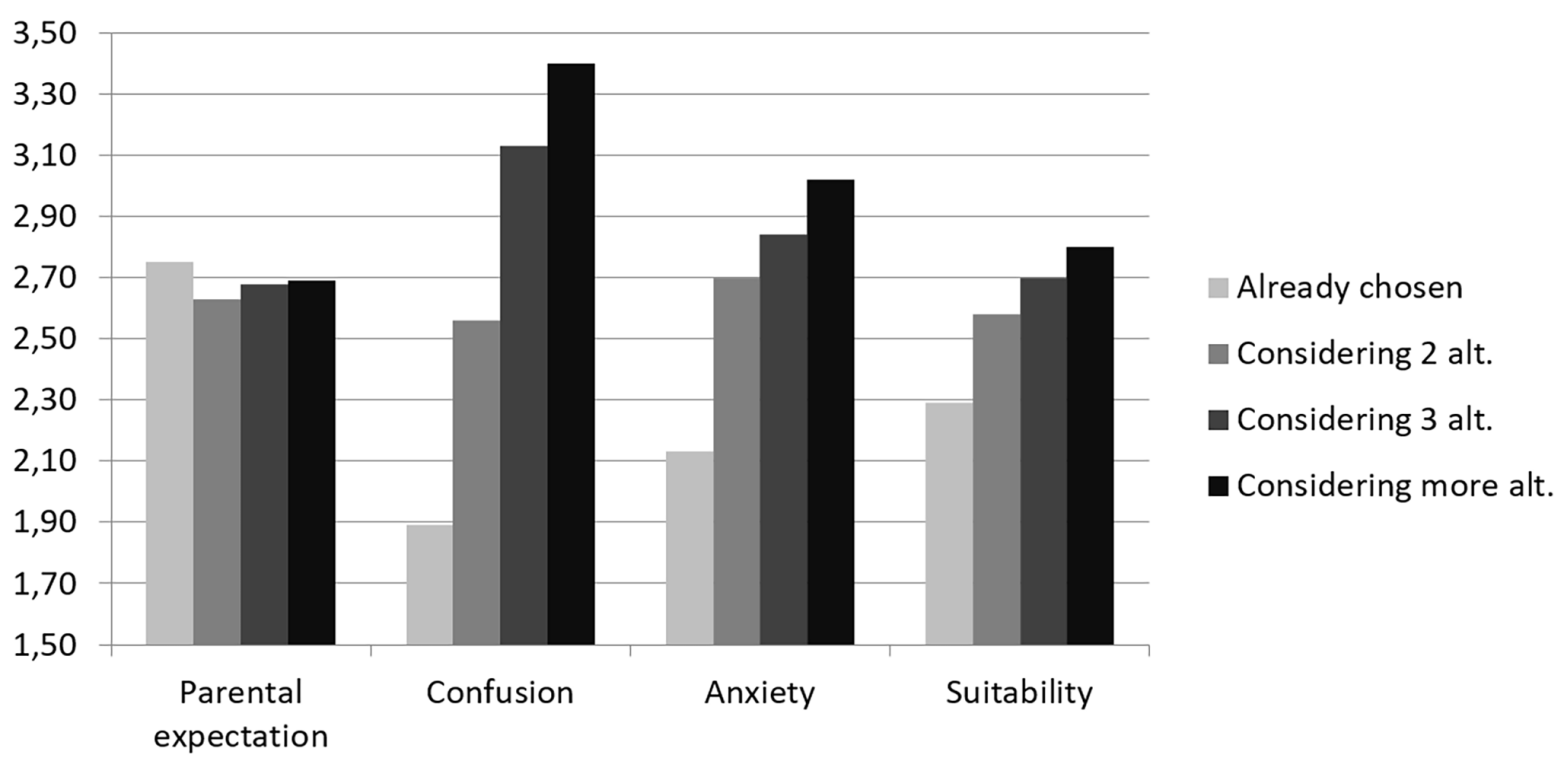
Mean PeCAS scales depending on the situation of the choice.

Students grouped according to the situation of the choice differed also in their perception of school choice difficulty. Just for parental expectations, ANOVA statistics were not significant, *F*(3) = 1.572; *p* = .194. At the opposite, confusion was the variable which differed more between the four groups, *F*(3) = 291.261, *p* < .001, followed by anxiety, *F*(3) = 64.218, *p* < .001, and suitability, *F*(3) = 34.606, *p* < .001 (Figure 2).

Gender differences were all significant, with women with greater scores in all the four scales, but they were very small in effect size, ranging from *d* = 0.11 for confusion to *d* = 0.33 for anxiety, as it was for the first study of Saka et al. (2008).

## Discussion

The objective of this study was to test the psychometric properties of PeCAS scale, a psychological questionnaire drawn to identify the perception of difficulties related to the choice of upper secondary school for students of the third year of middle school, and it demonstrated to possess very good psychometric features both for reliability and validity.

Confirmatory factor analysis confirmed the validity of the theorised structure, consisting of 4 scales, in part related to each other, and each one composed of 4 items. Also, reliability was good for all scales.

To test concurrent validity, the Exploration of Vocational Issue Scale (Kracke, 1997) was translated and validated in Italian, adapting it to school choices and for a very young target. This study confirmed the mono-factorial structure and showed internal consistency in line with the original research. The presence of significant correlations between each scale of PeCAS and the Exploration of Vocational Issue Scales proved a relationship between variables. Their modest intensity, on the other hand, showed that the five scales do not overlap and that, therefore, it is suggested to use both to understand the condition of students better.

### Limitations and Future Research Directions

The main limit of this study lies in the fact that the process of choosing can evolve differently for students and, consequently, their perception of difficulty. Subsequent studies should assess the progress of the perception of difficulty by making more administrations in the period from the beginning of the school’s activities of the last year at the time of formalisation of choice. If on the one hand one of the strengths is its simplicity, on the other hand, it is not wide enough to examine, in-depth, the specific difficulties a student can encounter. So, the use of a supplementary questionnaire, like the CDDQ (Gati, Krausz, & Osipow, 1996) is recommended to understand in more detail the reasons for which a specific student feels difficulty and to be able to proceed with a personalised counselling intervention more focused on his/her needs.

Even if the structure of the scale and the drafting of the items were inspired to several international questionnaires tested on different samples, this study was led only in one country, and students from just one type of school participated. Future studies should test its validity also on students involved in choosing different types of schools and college courses in different countries.

Future studies could also verify if parental career support (Boerchi & Tagliabue, 2018) and peer support (Muscarà et al., 2018) can play a role in school choice considering the mediation role of parental expectation. Moreover, other researchers could further indeep if career decision difficulties are associated with personality traits (Martincin & Stead, 2015) and emotional intelligence (Di Fabio et al., 2013).

### Implications for Career Counseling Practice

The simplicity of the instrument, both for compiling and interpreting the results, allows teachers, career counsellors, and school psychologists to use it directly to identify students who are experiencing more difficulties in choosing the upper secondary school or the university. This scale may also help them to implement pre-screening on a large number of students to plan guidance projects and to assess the effectiveness of school guidance support.

Moreover, it can be very useful for researchers because the limited number of items that compose the questionnaire make it suitable to be inserted in questionnaires composed of a large number of scales, reducing the risk of overwhelming respondents and, as a consequence, the risk of reducing the reliability of the data.
